# A case report: New-onset refractory status epilepticus in a patient with *FASTKD2*-related mitochondrial disease

**DOI:** 10.3389/fneur.2022.1063733

**Published:** 2023-01-11

**Authors:** Alexandra Astner-Rohracher, Matthias Mauritz, Markus Leitinger, Fabio Rossini, Gudrun Kalss, Caroline Neuray, Elisabeth Retter, Saskia B. Wortmann, Melanie T. Achleitner, Johannes A. Mayr, Eugen Trinka

**Affiliations:** ^1^Department of Neurology, Neurocritical Care, and Neurorehabilitation, Christian Doppler University Hospital, Centre for Cognitive Neuroscience, Paracelsus Medical University, Salzburg, Austria; ^2^Neuroscience Institute, Christian Doppler University Hospital, Centre for Cognitive Neuroscience Paracelsus Medical University Hospital, Salzburg, Austria; ^3^Neurology Practice, Tamsweg, Austria; ^4^University Children's Hospital, Paracelsus Medical University, Salzburg, Austria; ^5^Department of Pediatrics, Radboud Center for Mitochondrial Medicine, Amalia Children's Hospital, Radboudumc, Nijmegen, Netherlands; ^6^Karl Landsteiner Institute for Neurorehabilitation and Space Neurology, Salzburg, Austria; ^7^Department of Public Health, Health Services Research and Health Technology Assessment, UMIT–University for Health Sciences, Medical Informatics and Technology, Hall in Tirol, Austria

**Keywords:** new-onset refractory status epilepticus (NORSE), *FASTKD2* mutation, genetic epilepsies, mitochondrial disease, drug-resistant epilepsy

## Abstract

**Objectives:**

New-onset refractory status epilepticus (NORSE) is associated with high morbidity and mortality. Despite extensive work-up, the underlying etiology remains unknown in 50% of affected individuals. Mitochondrial disorders represent rare causes of NORSE. Biallelic variants in *FASTKD2* were reported as a cause of infantile encephalomyopathy with refractory epilepsy.

**Case description:**

In the study, we report a previously healthy 14-year-old with a new, homozygous *FASTKD2* variant presenting with NORSE. Following a seizure-free period of 7 years, he experienced another super-refractory SE and subsequently developed drug-resistant focal epilepsy, mild myopathy, optic atrophy, and discrete psychomotor slowing. Structural MRI at the time of NORSE showed right temporo-parieto-occipital FLAIR hyperintensity and diffusion restriction, with extensive right hemispheric atrophy at the age of 22 years. Whole-exome sequencing revealed a novel homozygous loss of function variant [c.(1072C>T);(1072C>T)] [p.(Arg358Ter);(Arg358Ter)] in *FASTKD2* (NM_001136193), resulting in a premature termination codon in the protein-coding region and loss of function of FASTKD2. Oxidative phosphorylation (OXPHOS) in muscle and skin fibroblasts was unremarkable.

**Conclusion:**

This is the first case of a normally developed adolescent with a new homozygous loss of function variant in *FASTKD2*, manifesting with NORSE. The phenotypical spectrum of FASTKD2-related mitochondrial disease is heterogeneous, ranging from recurrent status epilepticus and refractory focal epilepsy in an adolescent with normal cognitive development to severe forms of infantile mitochondrial encephalopathy. Although mitochondrial diseases are rare causes of NORSE, clinical features such as young age at onset and multi-system involvement should trigger genetic testing. Early diagnosis is essential for counseling and treatment considerations.

## Introduction

New-onset refractory status epilepticus (NORSE) is defined as refractory status epilepticus (SE) in individuals without previous history of epilepsy and no identification of an underlying cause within 72 h (1). An association with preceding febrile illness is common and even required in the subcategory of febrile infection-related epilepsy syndrome (FIRES). NORSE is associated with high morbidity and mortality, and the outcome strongly depends on the underlying etiology (2). Standardized diagnostic work-up is performed to identify structural, toxic, metabolic, or inflammatory causes (3–6) with infectious and autoimmune etiologies as the leading cause (7, 8). However, in up to 50% of patients, no etiology can be identified (8) (cryptogenic NORSE).

Mitochondrial diseases (MDs) are rare causes of SE (9). Currently, disease-causing variants in over 300 genes located in both the mitochondrial and nuclear DNA are known to be associated with MDs, resulting in heterogeneous phenotypes (9).

Biallelic variants in *FASTKD2*, encoding the protein Fas-activated serine/threonine kinase domain-containing protein-2, localizing to the inner mitochondrial matrix, have been reported in six individuals and represent a rare cause of infantile encephalomyopathy with refractory epilepsy and/or status epilepticus (10–12). In this study, we report the first case of a normally developed adolescent with a new homozygous loss of function variant in *FASTKD2*, manifesting with NORSE.

## Case description

**Figure 1:**
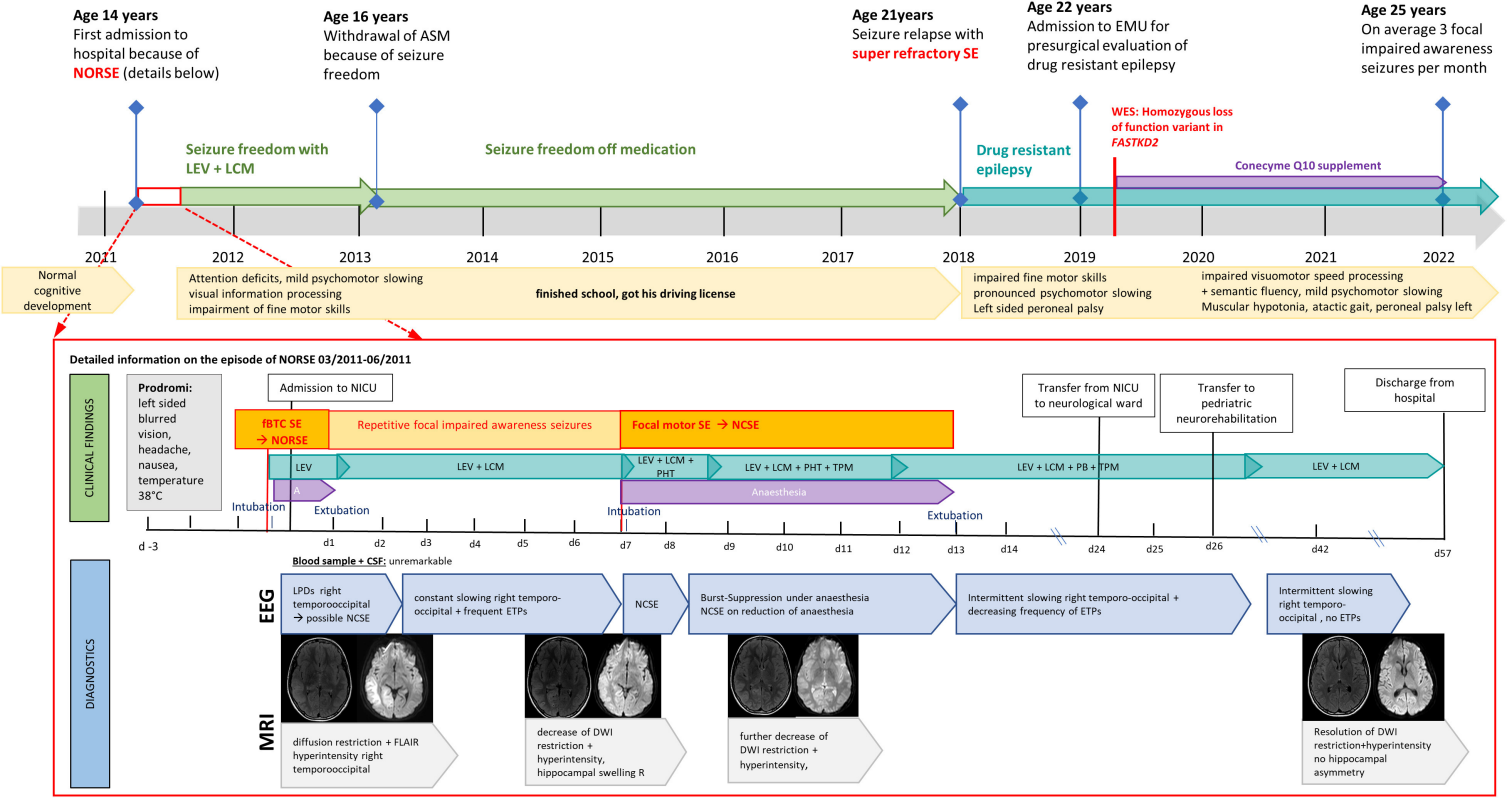
Diagnostic findings in a patient with FASTKD2 related mitochondrial disease presenting with new onset refractory status epilepticus (NORSE).

**Figure 2:**
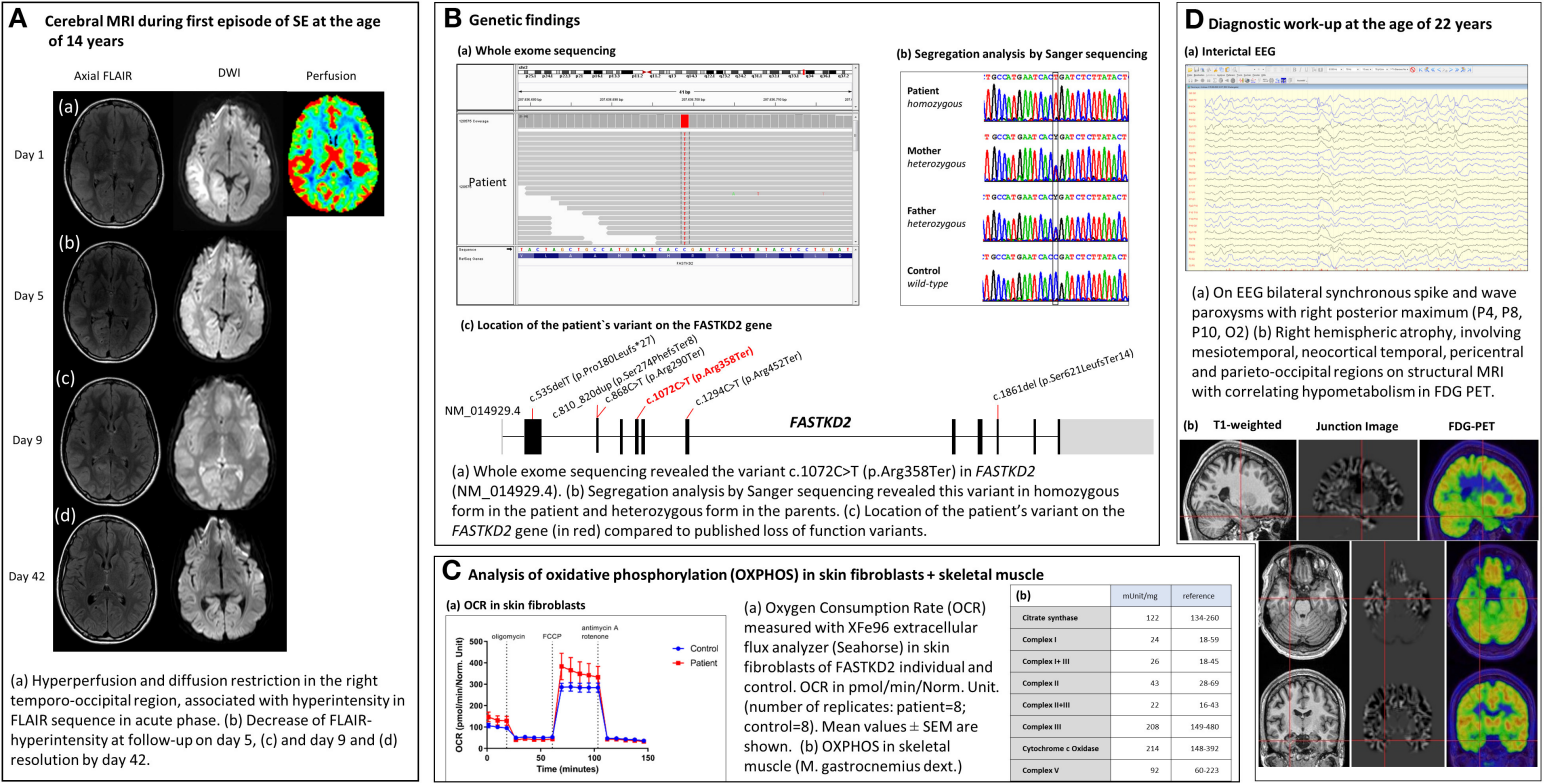
Timeline: Clinical course of a patient with FASTKD2 related mitochondrial disease.

The male patient was born as the second child to Caucasian parents with no known consanguinity or medical concerns after an uneventful pregnancy. His early motor development was unremarkable; he could walk at the age of 12 months and was athletic without rapid exhaustion during sports. In primary school, his fingers were observed in a peculiar positioning while writing, and his parents noted a “sloppy” gait and mild joint hypermobility, which were not further investigated. His performance at school was unremarkable. At the age of 14 years, he presented with NORSE at a district hospital in Salzburg, Austria (Figure 1). Three days before admission, he complained of left-sided blurred vision, headache, nausea, and high temperature of up to 38 degrees Celsius, qualifying the episode as FIRES. On the day of admission, his parents observed unsteady gait and psychomotor slowing for several hours, preceding a focal to bilateral tonic–clonic SE with head and eye deviation to the left. Administration of intravenous benzodiazepines (lorazepam, LZP 4 mg, diazepam, and DZP 16 mg) and antiseizure medication (ASM) (levetiracetam, LEV 2 g) led to the cessation of motor activity, but the impairment of consciousness persisted. The patient was intubated and transferred to the neurological intensive care unit (NICU) at the Paracelsus Medical University Hospital Salzburg, Austria. On arrival, he was comatose under anesthetic treatment without ictal motor activity or gaze deviation. Neurological examination revealed a subtle deformity of both feet with bilateral pes cavus. Acute brain MRI performed on arrival showed right temporo-occipital diffusion restriction, hyperperfusion, and hyperintensity in the FLAIR sequence (Figure 2). EEG concordantly revealed fluctuating lateralized periodic epileptiform discharges (LPEDs) over the same region, compatible with possible NCSE (13).

Initial laboratory testing and analysis of cerebrospinal fluid (CSF) identified no pathologies. Lactate levels in CSF and serum were within normal limits. Antiviral treatment with acyclovir and immunotherapy (immunoglobulins and corticosteroids) was started on day 1, but further extensive work-up of CSF revealed no infectious cause. Intravenous antiepileptic seizure medication (ASM) with LEV was established, and the patient could be weaned and extubated after 12 h. Thereafter, he presented with psychomotor slowing, slight anisocoria with mydriasis on the left side, and reported blurred vision. Because of repetitive focal impaired awareness seizures with head and eye deviation to the left, accompanied by clonic jerking on the left side, ASM was intensified (add-on lacosamide, LCM). Repeated EEG studies showed continuous slowing over the right posterior quadrant with frequently intermittent epileptiform discharges. MRI follow-up on day 5 demonstrated decreasing diffusion restriction and FLAIR hyperintensity in the right temporo-occipital region with mild right hippocampal swelling (Figure 2). Neural antibodies (anti-NMDA-R; anti-AMPA-R, anti-VKCC; LGI1/VGKC, anti-Yo, anti-Ri, anti-Hu, anti-CV2, anti-GABA-A/B, anti-MAG, and anti-Tr), and anti-thyroid antibodies (thyroperoxidase-TPO, thyrotropin-receptor-TRAK, thyroglobulin-TAK) were negative, as were mitochondrial diagnostics, including Sanger Sequencing for m.3243A>G (“MELAS”), m.8344A>G (“MERFF”) mitochondrial DNA point mutations, and of the entire *POLG* and *FXN* (Friedreich's ataxia) genes.

On day 7, the patient developed another focal motor SE with the impairment of consciousness and left-sided clonic, resistant to benzodiazepines and IV ASMs (LEV, LCM, phenytoin, and PHT). EEG showed lateralized periodic epileptiform discharges over the right hemisphere, with spatiotemporal evolution consistent with NCSE. Anesthetic treatment with thiopental was re-established with clinical and electrographic seizure recurrence on the withdrawal of anesthesia. SE persisted despite anesthetic treatment (switch to midazolam/ketamine), immunotherapy, high-dose magnesium, and intensified IV ASM (add-on topiramate, switch from PHT to phenobarbital). After 5 days, SE ceased on day 13, and the patient was gradually weaned from anesthetics. Thereafter, he showed slight ataxia, incomplete hemianopia to the left, vertical oscillatory nystagmus, slurred speech, and psychomotor slowing. He remained seizure free under high-dose ASM polytherapy (LEV, LCM, TPM, and PB), and neurological deficits improved gradually. MRI follow-up on day 9 showed a further decrease in diffusion restriction and FLAIR hyperintensity in the right temporo-occipital region (Figure 2). EEG gradually improved with persistent slowing over the right posterior quadrant but decreasing frequency of epileptiform discharges. Visual fields were normal, but vertical oscillatory nystagmus and discrete vertical palsy persisted. The patient had pronounced deficits in all cognitive domains, which gradually improved and were close to the lower normal age-adjusted range 3 months after discharge (see Supplementary material). ASMs were tapered down, and the patient remained seizure-free under dual therapy (LEV and LCM). MRI follow-up on day 42 demonstrated a resolution of diffusion restriction and FLAIR hyperintensity in the right hemisphere without hippocampal asymmetry. Intermittent slowing over the right posterior quadrant without epileptiform discharges persisted on EEG. Eleven weeks after admission, he was discharged from hospital with mild residual impairment of fine motor skills and attention deficits. Despite extensive diagnostic work-up, no underlying cause for the patient's new-onset epilepsy first manifesting with NORSE could be identified. ASMs were withdrawn after 2 years of seizure freedom; he finished school and got his driving license after 5 years of seizure freedom off medication.

Aged 21 years, after 7 years of seizure freedom, the patient relapsed with a super-refractory bilateral tonic–clonic SE treated at a community hospital in Carinthia, Austria (Figure 1). He developed a propofol infusion syndrome and septic multiorgan failure, treated with high-dose catecholamines, intermittent continuous veno-venous hemodialysis, and broad-spectrum antibiotics. SE was terminated after more than 6 weeks with gradual weaning from the ventilator after 2 months. He was discharged from hospital 3 months after admission with residual left-sided peroneal palsy, impaired fine motor skills, and pronounced psychomotor slowing.

Subsequently, the patient developed drug-resistant epilepsy with focal onset aware and impaired awaresse seizures despite high-dose polytherapy (LEV 4500 mg per day, LCM 1200 mg per day, and PHT 300 mg per day) and was admitted to our epilepsy-monitoring unit for presurgical evaluation aged 22 years (Figure 1).

## Diagnostic assessment

At the time of admission, the patient (22 years) presented with mild psychomotor slowing, discrete myopathy, spastic ataxic gait, and peroneal palsy on the left. Ictal and interictal EEG showed bilateral synchronous sharp waves with a maximum over the right posterior temporal to parieto-occipital head region. Structural MRI according to the in-house epilepsy protocol revealed an extensive right hemispheric atrophy involving mesiotemporal, neocortical temporal, pericentral, and parieto-occipital regions with correlating hypometabolism in fluorodeoxyglucose (FDG)-positron emission tomography (PET) (Figure 2).

Because of the multisystemic nature of the disease, mainly involving the central nervous system, muscles, and eyes, an MD was suspected. ECG detected a right-bundle branch block, and echocardiography was unremarkable. Baseline lactate levels in serum and CSF were normal, and bicycle ergometry detected no lactate increase during exercise. Abdominal ultrasound, liver and renal function were unremarkable. Ophthalmological examination showed slight bilateral optic atrophy. Muscle MRI revealed hyperintensity and atrophy in both gastrocnemii muscles with right-sided predominance. Electromyography showed signs of discrete myopathy.

Oxidative phosphorylation (OXPHOS) was evaluated in fresh muscle and skin fibroblasts (14) (Figure 2). Evaluation of OXPHOS in muscle (M. gastrocnemius right) revealed normal activities of the respiratory chain complexes I [24 mUnit/mg (18–59 mUnit/mg)], II [43 mUnit/mg, (28–69 mUnit/mg)], III [208 mUnit/mg, (149–480 mUnit/mg)], IV (cytochrome-C-oxidase) [214 mUnit/mg, (148–392 mUnit/mg)], V [92 mUnit/mg, (60–223 mUnit/mg)], and citrate synthase [122 mUnit/mg, (134–260 mUnit/mg)]. Oxygen consumption rate (OCR) was measured by Seahorse XFe96 analyzer in cultivated skin fibroblasts of the FASTKD2 individual and control. No decrease in either basal or maximal respiration could be detected (Figure 2).

Considering the patient's medical history and clinical findings, genetic testing was extended (Figure 2). Whole-exome sequencing from leucocyte-derived DNA was performed using a SureSelect Human All Exon V6 kit (Agilent). The coding regions were enriched, followed by sequencing as 100-bp paired-end runs on a HiSeq 4,000 (Illumina). Reads were aligned to the human reference genome (UCSC Genome Browser build hg19) using the Burrows–Wheeler Aligner (v.0.7.5 a) (15). Single-nucleotide variants, small insertions, and deletions were detected with SAMtools (version 0.1.19). Based on the assumption of autosomal recessive inheritance, variants were prioritized with a minor allele frequency of < 0.1%, and *de novo* variants were prioritized with a minor allele frequency of < 0.01%. As a result of this, we discovered a new homozygous loss of function *FASTKD2* variant [c.(1072C>T);(1072C>T)] [p.(Arg358Ter);(Arg358Ter)]. For confirmation and investigation of its segregation, the *FASTKD2* variant was investigated by targeted Sanger sequencing using the following forward primer 5'-CAGCACAAGACCCTGTCTCA-3' and reverse primer 5'-CTGGAGGTCTTTGCAGGACT-3'. The new *FASTKD2* variant is a non-sense mutation, resulting in the introduction of an in-frame premature termination codon (PTC) into the protein-coding gene sequence and, subsequently, loss of the function of FASTKD2 (NM_001136193). Both parents are heterozygous carriers of this variant (Figure 2). According to the ACMG criteria, (16) the FASTKD2 variant is classified as “pathogenic” (score: 11, PVS1: very strong, PP5: moderate, and PM2: supporting). In the database ClinVar, this variant has been reported as “likely pathogenic” (allele ID: 1675474).

Based on these findings, genetic counseling at the Department of Neuropediatrics was performed, and treatment options were discussed. As no causal therapy is available for this rare mutation, lifestyle modifications with a structured daily routine, a healthy diet, aerobic sports, and sufficient periods of rest were emphasized. Coenzyme Q10 supplement and ketogenic diet were established, but the latter was stopped as no improvement in seizure frequency could be achieved, and the diet was not well-tolerated by the patient.

The patient is currently aged 25 years and suffers from an average of three focal onset aware and impaired awareness seizures per month, despite ongoing high-dose ASM polytherapy (LEV 3,250 mg per day, PHT 300 mg per day, LCM 600 mg per day, and PER 12 mg per day). No focal to bilateral tonic–clonic seizures or SE occurred since diagnosis. Neurological examination and neuropsychological assessment showed no deterioration of cognitive function or neurological deficits at the last follow-up, with persistent mild psychomotor slowing, impairment of semantic fluency, and visuomotor processing speed.

## Discussion

In this study, we describe a patient with a new biallelic homozygous *FASTKD2* variant associated with NORSE and recurrent RSE. The discovered *FASTKD2* variant is a non-sense mutation, resulting in the introduction of an in-frame PTC into the protein-coding gene sequence and, subsequently, loss of function of FASTKD2. FASTKD2 is a protein located in the inner mitochondrial matrix and is presumably involved in mitochondrial ribosomal assembly, mtRNA stabilization, and translation (17, 18). Isolated complex IV deficiency and decreased COX staining were detected in one patient with FASTKD2-related MD (11). FASTKD2 and helicase DDX28 are also required for 16S rRNA-binding during ribosome assembly in mitochondria (17), and loss of function variants in *FASTKD2* were associated with the impairment of OXPHOS complexes I–IV and ATPase (17, 18). *In vitro* studies of immortalized lymphocytes of two individuals with FASTKD2-related MD (12) detected reduced 16S rRNA expression and decreased activity of OXPHOS-complexes-containing-mtDNA subunits, suggesting that defective mtRNA translation might lead to multiple OXPHOS complex deficiency other than complex IV (12). In our patient, as in one other patient presenting with a late onset comparatively mild phenotype (10), OXPHOS was unremarkable in skeletal muscle and skin fibroblasts, suggesting a possible correlation between disease severity and alterations of OXPHOS.

To date, only six patients with FASTKD2-related MD have been published (summarized in Table 1) (10–12). The first two reported cases were siblings from consanguineous parents with early onset severe encephalomyopathy and refractory epilepsy (11). The underlying mutation was a homozygous nonsense mutation in the *KIAA0971* gene, encoding for the FASTKD2 protein. A biochemical analysis revealed highly decreased COX function in the muscle mitochondria of one patient and lymphocytes of the other. A completely different phenotype with late disease onset at the age of 15 years and MELAS-like clinical presentation without developmental delay was described in a third patient with FASTKD2-related MD (10). A compound heterozygous mutation (p.R205X and p.L255P) in the *FASTKD2* gene was discovered in this patient, and no alterations of OXPHOS were detected by the analysis of skeletal muscle. The latest report on FASTKD2-related MD described three patients with infant-onset encephalomyopathy with moderate neurodevelopmental delay. Three different novel *FASTKD2* mutations (c.808_809insTTTCAGTTTTG, homoplasmic mutation c.868C>T, and heteroplasmic mutation c.1859delT/c.868C>T) were discovered in these patients, all of them leading to truncated FASTKD2 variants, lacking the C-terminus RNA-binding domain. A mitochondrial function analysis in immortalized lymphocytes revealed multiple OXPHOS deficiencies not isolated to complex IV in two of those patients.

**Table 1:** Comparison of published cases with FASTKD2-related mitochondrial disease (FASTKD2-MD).

	**Patient 1** **Ghezzi et al. (11)**	**Patient 2** **Ghezzi et al. (11)**	**Patient 3** **Yoo et al. (10)**	**Patient 4** **Wei et al. (12)**	**Patient 5** **Wei et al. (12)**	**Patient 6** **Wei et al. (12)**	**Patient 7** **Astner-Rohracher et al. (19)**
*FASTKD2* mutation	Homozygous non-sense mutation in the KIAA0971 gene p.R416X + p.R416X	Homozygous non-sense mutation in the KIAA0971 gene p.R416X+ p.R416X	Compound heterozygous mutations p.R205X (c.613C>T) + p.L255P (c.764T>C)	Homozygous mutation at p.L270fs^*^11 (c.808_809insTTTCAG TTTTG)	Homozygous mutation p.R290 (c.868C>T)	Two compound heterozygous mutations at c.1859delT/c.868C>T and p.S621Lfs^*^14/p.R290	Homozygous mutation p.[Arg358Ter]; [Arg358Ter] (c.[1072C>T]; [1072C>T])
Ethnics/nationality	Bedouin (Israel) sister	Bedouin (Israel) Brother	Korean	Chinese	Chinese	Chinese	Austrian
Consanguinity	Yes (first degree cousins)	Yes (first degree cousins)	No	No	Yes	No	No
Sex	Female	Male	Male	Female	Female	Female	Male
Age at disease onset	7 months	1 year	15 years	6 months	22 months	1 year	14 years
First symptom	Fever associated seizure	Fever associated subacute neurological deterioration (Muscle hypotonia, extrapyramidal movements left>right)	Generalized tonic clonic seizure	Axial hypotonia + dyskinesia	Seizure	Seizure	Focal to bilateral tonic clonic seizure -> refractory SE (NORSE)
Developmental delay	Delayed development from age 7 months, at age 14 y: follows simple commands, 20 words vocabulary, can sit, is not able to walk	Deterioration of neurological development from age 1 year, at 4 years bed-ridden with neither communication nor any voluntary activity.	No	Delayed motor development, able to sit at the age of 9 months, walks at the age of 3	Yes, no further information given	Delayed development from the beginning, unable to sit until 7 months and walk until 22 months of age	No
Status epilepticus (SE)	No	Repeated SE	1st SE at age 18 y, 2nd SE at age 26 y	No	No epilepsy	No	Refractory SE at age 14 y and age 21 y
Clinical manifestations	Developmental delay, Myoclonic and gelastic seizures, optic atrophy, spastic left-sided hemiparesis	Developmental delay, refractory seizures with repeated SE, optic atrophy, muscle hypotonia, extrapyramidal symptoms	Stroke-like episode with visual field deficit left, epilepsy, Bilateral optic atrophy	Developmental delay, axial hypotonia, dyskinesia	Dyskinesia, unconscious shaking of hands, occasional convulsions at 3 years	Nystagmus, hypotonia, slurred speech, diminished deep tendon reflexes in the lower limbs.	Refractory status epilepticus twice, drug-resistent focal epilepsy, mild psychomotor slowing, myopathy, spastic atactic gait
Brain MRI	MRI at age 7 months: Generalized Symmetric atrophy CT at age 5y: Right hemispheric atrophy	MRI at age 1 y: Hyperintensity left nucleus caudatus, globus pallidus, and crus cerebri CT at age 2.5 y: Generalized atrophy, more pronounced on the left basal ganglia, bilateral dilatation of ventricles + basal cysternae	Right occipital lobe infarction	MRI at age 14 months:high T2 signal intensity in bilateral globus pallidus, medulla oblongata, and mesencephalon	MRI at age 9 years:bilateral symmetrical hyperintensity in globus pallidus	MRI at age 1 year 8 months:Brain atrophy, bilateral symmetrical hyperintensity signals in globus pallidus, putamen, and caudate nucleus MRI at age 2.5 years: T2 hyperintensity bilateral basal ganglia and cerebral atrophy	MRI at age 14 years: Diffusion restriction and FLAIR hyperintensity right temporo-occipital MRI at age 22 years: atrophy right temporo-parietal
EEG	Bilateral epileptiform discharge left > right	Right hemispheric attenuation + triphasic waves over left hemisphere	Slowing right hemisphere, sharp transients right parieto-occipital region	Not performed	Abnormal (not specified)	Not reported	Bilateral synchronous spike and wave with right posterior maximum
Lactate level	Serum 2.4–3.2 mM (normal < 1.8 mM)	CSF 3.8 mM (normal < 1.8 mM),	Serum 2.2 mM (normal < 1.6 mM)	Serum 3.4 mM (normal < 2.1 mM)	Normal: 1.9 mM (normal < 2.1 mM)	Serum 6.3 (normal < 2.1 mM)	Serum 2.2 mmol/l CSF 2.2 mmol/l (normal 1.1–2.2 mmol/l)
Abdominal sonography	Normal	Normal	Normal	Not performed, laboratory testing normal	Not reported	Not reported	Normal
Renal function	Normal	Normal	Normal	Normal	Not reported	Not reported	Normal
Specific therapy	Not reported	Not reported	Coenzyme Q10	Not reported	Not reported	Not reported	Coenzyme Q10
Echocardiography	Normal	Normal	Normal	Not reported	Hypertrophic cardiomyopathy, sinus tachycardia	Not reported	Normal
Optic nerve	Bilateral opticatrophy	Bilateral opticatrophy	Bilateral opticatrophy	Not reported	Not reported	Not reported	Bilateral mild atrophy of temporal fibers
Muscle biopsy	COX activity reduced to 21% of controls, other respiratory chain complexes: normal	Not performed	SDH, COX, mGT stain: normal	Not performed	Not performed	Not performed	COX, citrate synthase, respiratory chain complexes I, II, III, V: normal
Skin fibroblasts	Normal activity of MRC	Normal activity of MRC					OCR: no decrease of basal or maximal respiration
Immortalizedlym phozytes	Not performed	Decreased COX activity		16s-rRNA 30% lower compared to controls; 8.5-fold higher extracellular lactate generation	16s- rRNA 54% lower compared to controls; 4.3-fold higher extracellular lactate generation	Not performed	Not performed
Visual evoked potentials	Not performed	Not performed	Delayed	Not performed	Not performed	Not performed	Not performed

COX, cytochrome C oxidase; mGT, modified Gomori trichrome; MRC, mitochondrial respiratory chain complex; NORSE, new-onset refractory status epilepticus; OCR, oxygen consumption rate; SE, status epilepticus; SDH, succinate dehydrogenase.

In summary, the genetic and phenotypical spectrum of published cases with FASTKD2-related MD is highly variable, with age at disease onset ranging from 6 months to 15 years. Developmental delay was a leading symptom in all but one patient (10), ranging from moderate delay in early motor milestones to severe deterioration of psychomotor function with the inability to walk or speak following normal development (11). In contrast, psychomotor development was unremarkable in our patient, and no cognitive decline or progression of neurological symptoms was detected at the last follow-up. Muscular hypotonia (11, 12) and bilateral optic atrophy (10, 11) were common findings observed in three published cases as well as in our patient. Furthermore, different from our patient, elevated lactate levels in serum or CSF were measured in all previously published cases but one. Epileptic seizures with subsequent development of refractory epilepsy are the leading symptom in our patient and represented the first clinical symptom in five of six previously published cases. Recurrent SE was reported in two patients (10, 11); however, this is the first case manifesting with NORSE. In addition, involvement of basal ganglia with extrapyramidal symptoms and correlating hyperintensity in globus pallidus (3/6 bilateral; 1/6 unilateral) on brain MRI was described in half of the patients. Global brain atrophy on MRI was seen in three individuals, and one patient with early onset disease developed left-sided hemiparesis with concordant severe unilateral right hemispheric brain atrophy (11). Unilateral brain atrophy with posterior maximum was also found in our patient without focal neurological deficits. Cardiac involvement with hypertrophic cardiomyopathy and sinus tachycardia was detected in one patient with moderate early onset encephalomyopathy, whereas the cardiological work-up of our patient, as of the other published cases, revealed no pathologies.

The case we report here adds to the phenotypical spectrum of FASTKD2-related MD. Clinical presentation with late-onset disease manifesting with NORSE is unique. Biochemical findings and genetic profiles differ from previously reported cases. The discovered variant has not been described previously and, in contrast to other cases, no alterations of OXPHOS could be detected. However, due to tissue specificity, unremarkable findings in skeletal muscle and skin fibroblasts do not exclude alterations of OXPHOS in other tissue/organs. Furthermore, a correlation between disease severity and alterations in OXPHOS can be hypothesized. Altogether, this case emphasizes the heterogeneous phenotypical spectrum of MDs and further contributes to understanding the complexity of FASTKD2-related MDs.

Epilepsy is a common symptom of mitochondrial disease (20), but the underlying pathophysiological mechanisms leading to SE are incompletely understood. Bioenergetic failure with the subsequent collapse of ionic gradients leading to apoptotic cell death and oxidative stress with the overproduction of reactive oxygen species might play an important role in seizure perpetuation. However, the role of mitochondrial dysfunction in SE is more complex, including immune dysfunction and impaired mitochondrial dynamics (9). The pathophysiological mechanisms leading to NORSE in our patient can only be hypothesized. Even in the absence of evidence for the impairment of OXPHOS, bioenergetic failure, and oxidative stress are probably among the leading causes.

Despite the sparse literature relating NORSE to MD (8), clinical features such as seizures, optic atrophy, cardiomyopathy, increased serum or CSF lactate, and MRI abnormalities should raise suspicion of an underlying MD in individuals presenting with NORSE or new-onset complex epilepsies. Exome or genome-wide genetic testing, including both nuclear and mtDNA, should be considered even in the absence of other clinical findings. Identifying the exact (genetic) diagnosis is key for proper counseling and treatment considerations. A ketogenic diet seems promising for seizures in certain MD subtypes (21), and pathomechanism-based treatment options are increasingly available (22). To date, no targeted therapy is available for FASTKD2-MD. However, therapeutic strategies suppressing PTCs and restoring the deficient protein function show good results in other diseases (23) and might also be a promising approach in our case of FASTKD2-MD. Future research might enable tailored therapy that influences seizure control and disease progression in these patients.

Furthermore, frequently used drugs in the treatment of (NOR)SE, such as valproic acid (VPA), propofol, or thiopental, should be used with caution due to the increased risk of hepatic failure and propofol infusion syndrome in certain MD subtypes, especially VPA in POLG-related MD. In our patient, the earlier genetic diagnosis could have prevented the development of propofol infusion syndrome.

Future research and international collaborations and registries are needed, especially in these cases of rare and complex genetic epilepsies, to gain knowledge on clinical course, treatment response, and prognosis. This is essential to guide future treatment decisions and counseling of patients and their families.

## Data availability statement

The datasets presented in this article are not readily available because of ethical and privacy restrictions. Requests to access the datasets should be directed to the corresponding authors.

## Ethics statement

Ethical review and approval was not required for the study on human participants in accordance with the local legislation and institutional requirements. The patients/participants provided their written informed consent to participate in this study. Written informed consent was obtained from the individual(s) for the publication of any potentially identifiable images or data included in this article.

## Author contributions

AA-R contributed to the study concept, data acquisition, and drafting of the manuscript. MM, GK, and ER contributed to the data acquisition. FR, ML, SW, and JM contributed to the data acquisition and drafting of the manuscript. CN contributed to the drafting of the manuscript. MA contributed to data acquisition. ET contributed to the study concept and the drafting of the manuscript. All authors contributed to the article and approved the submitted version.
