# Supplementary material for: A case report: New-onset refractory status epilepticus in a patient with FASTKD2-related mitochondrial disease
Source: Front Neurol. 2023 Jan 11;13:1063733. doi: 10.3389/fneur.2022.1063733 (PMC9875587; doi:10.3389/fneur.2022.1063733)
Supplement: Supplementary file 1 [file Image_1.pdf]

**Supp. Fig 1.: Evolution of neuropsychological performance of a patient with FASTKD2-related mitochondrial disease**

| | **Age 14 years,** During admission at the time of NORSE | **Age 14 years,** Follow-Up 3 months after NORSE | **Age 22 years,** During presurgical evaluation |
|---|---|---|---|
| **COGNITION** | *WISC-IV* | *WISC-IV* | *WAIS* |
| Intelligence quotient | **56 [52-62] (normal 84-115) ; PR 0.2 (16-84)** | **81 [70-88] (84-115) ; PR 10.3** (16-84) | |
| Vocabulary | **PR 4.8* (16-84)** | PR 25.2 (16-84) | IQ 95 (*85-115)* |
| Visual Construction | **PR 0.3* (16-84)** | | IQ 90 (*85-115)* |
| Visual convergent thinking | **PR 0.7* (16-84)** | PR 25.2 (16-84) | IQ 90 (*85-115)* |
| **MEMORY** | *VLMT; DCS* | *VLMT,DCS* | *VLMT* |
| Verbal Memory | **PR < 5* (16-84)** | | IQ 85 (*85-115)* |
| Figural Memory | PR 30(16-84) | | IQ 103 (*85-115)* |
| **ATTENTION** | *TAP* | *TAP* | *TAP* |
| Divided attention | PR 21(16-84) | | IQ 85 (*85-115)* |
| Mental processing speed | **impaired** | **PR 5.5* (16-84)** | **IQ 55*** (*85-115)* |
| Alertness phasic | PR 66 (16-84) | | **IQ 73*** (*85-115)* |
| **EXECUTIVE FUNCTION** | *TL-3 , WISC-IV* | *TL-3, WISC-IV* | *Trail Making Test B, WAIS* |
| Working Memory visual | **PR 0.2*(16-84)** | **PR 11.5* (16-84)** | IQ 94 (*85-115)* |
| Working Memory verbal | | | IQ 90 (*85-115)* |
| Semantic fluency | | | **IQ 65*** (*85-115)* |
| Inhibition control | **PR < 1* (16-84)** | | **IQ 64*** (*85-115)* |
| Flexibility | average | | **IQ 55*** (*85-115)* |

*** Values below average**

DCS…diagnostics for cerebral injuries, PR… percentage range, TAP…Test of Attentional Performance, TL-3…tower of london test; VLMT…verbal learning and memory test, *WAIS… Wechsler Adult Intelligence Scale, WISC-IV (Wechsler Intelligence Scale for Children, 4th Edition)*
